# Prolonged *Plasmodium falciparum* Infection in Immigrants, Paris

**DOI:** 10.3201/eid1402.061475

**Published:** 2008-02

**Authors:** Eric D’Ortenzio, Nadine Godineau, Arnaud Fontanet, Sandrine Houze, Olivier Bouchaud, Sophie Matheron, Jacques Le Bras

**Affiliations:** * Centre Hospitalier Universitaire Bichat-Claude Bernard, Paris, France; † Centre Hospitalier Général Delafontaine, Saint-Denis, France; ‡ Institut Pasteur, Paris, France; §Université Paris Descartes, Paris, France; ¶ Centre Hospitalier Universitaire Avicenne, Bobigny, France; # Université Paris 13, Paris, France; ** Université Paris Diderot, Paris, France; 1 Current affiliation: Institut de Veille Sanitaire, Saint-Denis, Réunion Island, France

**Keywords:** Plasmodium falciparum, imported malaria, prolonged infection, traveler, immigrant, pregnancy, mefloquine, transfusion risk, dispatch

## Abstract

Few immigrant travelers have *Plasmodium falciparum* infections >2 months after leaving malaria-endemic areas. We conducted a case–control study to identify factors associated with prolonged *P. falciparum* infection in immigrant travelers. Results suggest that *P. falciparum* infection should be systematically suspected, even months after travel, especially in pregnant women and first-arrival immigrants.

Approximately 100 countries endemic for malaria are visited by 125 million international travelers yearly, and >30,000 contract imported malaria (*1*). In France, the number of imported cases of *Plasmodium falciparum* malaria was estimated to be 4,500 in 2004, with a median time of 10 days between departure from an area endemic for malaria and diagnosis (*2*). The duration of a *P*. *falciparum* infection in humans is generally believed not to exceed 12 months. Most epidemiologic studies show that few patients have malaria onset >2 months after returning from travel (*3*,*4*). Late occurrence of infection could have severe consequences if physicians do not relate symptoms suggestive of malaria to travel history. Another risk is transfusion-transmitted malaria from an asymptomatic carrier of *P*. *falciparum* trophozoites (*5*).

Cases of late occurrence of *P*. *falciparum* malaria have been reported (*6*–*9*), but risk factors are unknown. The objective of this study was to determine the incidence and identify factors associated with prolonged *P*. *falciparum* infection in immigrant travelers.

## The Study

A case–control study was conducted among patients with *P*. *falciparum* malaria diagnosed at Bichat-Claude Bernard and Saint-Denis Hospitals in Paris, France. Many African immigrants come to these hospitals. Participants traveled to or lived in an area endemic for malaria and had a *P*. *falciparum* infection during 1996–2005. The diagnostic criterion was *P*. *falciparum* trophozoites on a blood smear confirmed by the Centre National de Reference du Paludisme (CNRP) in Paris, without epidemiologic evidence of autochthonous, transfusion-transmitted, or occupational malaria. Case-patients had *P*. *falciparum* infections detected >59 days after their arrival in France. Controls had *P*. *falciparum* infections detected <30 days after their arrival. For each case-patient, 4 controls were matched by calendar year and hospital of diagnosis (70 cases and 280 controls). Data were collected from the CNRP database in which all cases are prospectively included and medical records are checked. We only considered immigrants (persons born in an area endemic for malaria and residing in France), which resulted in 61 case-patients and 197 controls. We distinguished first-arrival immigrants (persons who emigrated to France and never returned to areas endemic for malaria) from visiting friends and immigrant relatives (persons who traveled back to areas endemic for malaria after immigration to France).

Logistic regression was used to identify factors associated with prolonged *P*. *falciparum* infection and estimate odds ratios (ORs) and 95% confidence intervals (CIs). For multivariate analysis, variables with p values <0.25 were introduced into the model and removed after a backward stepwise approach, which resulted in only values with p<0.05 in the final model (except for age groups). Statistical analysis was performed by using Stata software version 8.2 (Stata Corporation, College Station, TX, USA).

During the 10-year period, 61 (2.3%) late infections occurred among 2,680 diagnosed *P*. *falciparum* malaria infections. The median diagnosis delay was 5 months (interquartile range 3–9 months). These infections included 10 patients (5 pregnant women, 2 HIV-positive patients, and 3 first-arrival immigrants) with clinical malaria >1 year after their arrival. Four of them, all pregnant women, had clinical malaria >3 years after their arrival. For the case–control study, 197 controls were compared with 61 case-patients (Figure). Table 1 shows the main characteristics of case-patients and controls. Case-patients were younger (median age 30.6 years vs. 34.5 years, p = 0.04) and more often female (54.1% vs. 38.1%, p = 0.03) than controls. The mean parasitemia level was lower for case-patients than for controls (0.6% vs. 1.4%, p = 0.04), including patients with 8 asymptomatic cases versus none of the controls (in these cases, diagnosis of malaria was made through systematic checking).

**Figure:**
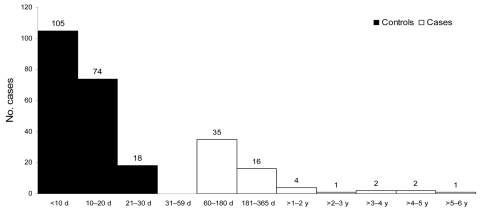
Delay in days or years between arrival in France and diagnosis of imported *Plasmodium falciparum* malaria, Bichat-Claude Bernard Hospital and Saint Denis Hospital, Paris, France, 1996–2005.

**Table 1:** Univariate analysis of factors associated with prolonged *Plasmodium falciparum* infection in 248 immigrant travelers*

Variable	Case-patients (n = 61), no. (%)	Controls (n = 197), no. (%)	OR (95% CI)	p value
Sex				
Female	33 (54.1)	75 (38.1)	1	
Male	28 (45.9)	122 (61.9)	0.52 (0.29–0.93)	0.03
Age, y				
<5	2 (3.3)	10 (5.1)	1	
5–14	5 (8.2)	18 (9.1)	1.39 (0.22–8.51)	
15–60	53 (86.9)	163 (82.7)	1.63 (0.34–7.66)	0.83
>60	1 (1.6)	6 (3.1)	0.83 (0.06–11.23)	
Origin				
Sub-Saharan Africa	55 (90.2)	181 (91.9)	1	
Comoros Islands	5 (8.2)	14 (7.1)	1.18 (0.41–3.41)	
Other	1 (1.6)	2 (1)	1.65 (0.15–18.5)	0.89
South America	0	1 (0.5)	NA	
Caribbean	0	1 (0.5)	NA	
India	1 (1.6)	0	NA	
First-arrival immigrant				
No	24 (39.3)	183 (92.9)	1	
Yes	37 (60.7)	14 (7.1)	20.15 (9.54–42.57)	<0.001
Region of malaria acquisition				
West Africa	32 (52.5)	132 (67)	1	
Central Africa	22 (36.1)	44 (22.3)	2.06 (1.09–3.92)	0.03
East Africa	1 (1.6)	2 (1)	2.06 (0.18–23.46)	
Comoros Islands	5 (8.2)	18 (9.1)	1.15 (0.4–3.32)	
Other	1 (1.6)	1 (0.5)	4.12 (0.25–67.74)	
Chemoprophylaxis				
No	50 (82)	118 (59.9)	1	
Yes	9 (14.8)	71 (36)	0.3 (0.14–0.65)	0.002
Unknown	2 (3.2)	8 (4.1)	NA	
Prophylaxis with mefloquine				
No	56 (91.8)	186 (94.4)	1	
Yes	3 (4.9)	3 (1.5)	3.32 (0.65–16.92)	0.15
Unknown	2 (3.3)	8 (4.1)	NA	
Antimalarial self-medication				
No	55 (90.2)	171 (86.8)	1	
Yes	5 (8.2)	15 (7.6)	1.04 (0.36–2.98)	0.95
Unknown	1 (1.6)	11 (5.6)	NA	
Men	28 (45.9)	122 (61.9)	1	
Nonpregnant women	16 (26.2)	69 (35)	1.01 (0.51–2)	
Pregnant women	17 (27.9)	6 (3.1)	12.35 (4.46–34.14)	<0.001
HIV status				
Negative	22 (36.1)	42 (21.3)	1	
Positive	12 (19.7)	6 (3.1)	3.82 (1.26–11.56)	0.02
Unknown	27 (44.3)	149 (75.6)	NA	
Symptomatic malaria				
No	8 (13.1)	0 (0)	1	
Yes	53 (86.9)	197 (100)	0.3 (0.25–0.47)	<0.001
Parasitemia†				
High	2 (3.3)	18 (9.1)	1	
Low	59 (96.7)	179 (90.9)	1.61 (0.53–4.9)	0.4

*OR, odds ratio; CI, confidence interval; NA, not applicable. †Parasitemia (parasitized erythrocytes) was considered high if >4% and low if <4% by World Health Organization criteria.

Among immigrant travelers, 3 groups had a higher risk for prolonged *P*. *falciparum* infection: pregnant women, first-arrival immigrants, and HIV-positive patients. A total of 27.9% (n = 17) of the patients were pregnant women, with a median (range) age of 22 (16–36) years. All were of African origin and had become pregnant in France; 10 (58.8%) were in their second trimester, 5 (29.4%) were in their third trimester. First-arrival immigrants were younger than other patients (mean age 26.2 vs. 37.6 years, p = 0.001). All patients were of African origin except for 1 Indian man. HIV infection was associated with prolonged infection, but HIV status was not introduced into the final model because of missing data. Although chemoprophylaxis with chloroquine-proguanil was less common among case-patients than controls (8.5% vs. 21.2%, p = 0.03), the reverse was seen, although not significantly, with mefloquine use (4.9% vs. 1.5%, p = 0.15). Multivariate analysis (Table 2) showed that factors positively and independently associated with prolonged *P*. *falciparum* infection in immigrant travelers were being a first-arrival immigrant (OR 22.93, 95% CI 9.74–53.96, p<0.001), being a pregnant woman (OR 4.21, 95% CI 1.13–15.77, p = 0.03), and mefloquine prophylaxis (OR 11.55, 95% CI 2.06–64.78, p<0.005).

**Table 2:** Factors independently associated with prolonged  *Plasmodium falciparum* infection in 248 immigrant travelers*

Variable	OR (95% CI)*	p value
Age, y		
<5	1	
5–14	1.45 (0.15–13.74)	
15–60	1.72 (0.25–12)	
>60	3.04 (0.16–56.25)	0.45
First-arrival immigrant		
No	1	
Yes	22.93 (9.74–53.96)	<0.001
Men	1	
Nonpregnant women	0.67 (0.28–1.59)	
Pregnant women	4.21 (1.13–15.77)	0.03
Use mefloquine		
No	1	
Yes	11.55 (2.06–64.78)	0.005

*OR, odds ratio; CI, confidence interval.

We also observed cases of malaria in a 26-year-old Caucasian man and a 2-year-old African girl who were hospitalized with diagnosis delays of 221 days and 127 days, respectively. The man was a French expatriate who lived in Madagascar for 2 years and took regular chloroquine-proguanil prophylaxis. He was hospitalized 7 months after his return with severe *P*. *falciparum* malaria (impaired consciousness) and responded to treatment. The girl had traveled in Mali for 2 weeks and took regular chloroquine-proguanil prophylaxis. She was hospitalized 4 months after her return with uncomplicated *P*. *falciparum* malaria occurring concomitantly with a *Salmonella* spp. infection that had been treated 1 week earlier with ceftriaxone.

## Conclusions

Three independent factors were positively associated with prolonged *P*. *falciparum* infection: being a first-arrival immigrant, being a pregnant woman, and taking mefloquine prophylaxis. The first 2 factors most likely reflect partial control of parasitemia by acquired immunity. Persons living in areas with high transmission of malaria acquire this immunity during childhood. In these areas, *P*. *falciparum* infections in adults are mostly asymptomatic with transient low parasitemia levels (*10*). Chronic asymptomatic carriage of *P*. *falciparum* helps prevent symptomatic malaria attacks (*11*). In a previous study, 29% of asymptomatic Liberian children had detectable *P*. *falciparum* 4 weeks after immigration to the United States (Minnesota) (*12*). We postulate that many first-arrival immigrants are asymptomatic *P*. *falciparum* carriers upon their arrival in France. Their immunity probably prevents clinical symptoms for a few months, but in the absence of reinfections their immunity would decrease and symptoms would occur. In some cases, *P*. *falciparum* infections may not be the cause of illness when patients come to a hospital.

Several immunologic mechanisms have been suggested to explain late manifestations of *P*. *falciparum* malaria in pregnant women. A decrease in immunity during pregnancy could be one explanation (*10*). Other authors have suggested that antigenic variability could be responsible for impaired control of parasitemia (*13*). The role of mefloquine prophylaxis in delayed onset of malaria has been suggested (*14*). We found a positive association between mefloquine use and prolonged *P*. *falciparum* infection, but this drug was seldom used by our study group. This association is probably caused by the long half-life of mefloquine (>3 weeks).

This study also highlights the risk for blood transfusion–transmitted malaria, a rare but serious complication. Mungai et al. (*15*) reported 32 cases of transfusion-transmitted *P*. *falciparum* malaria in the United States during 1963–1999 (mortality rate 18.8%). Current US guidelines recommend obtaining a thorough travel history and deferring blood donation if potential donors have emigrated from areas endemic for malaria in the preceding 3 years. However, this measure may not prevent transmission if *P*. *falciparum* is present for >3 years (as in 4 pregnant women in our study). In France, systematic serologic analysis for *Plasmodium* spp. in blood donors born in areas endemic for malaria was implemented in 2002 (*5*).

Our findings suggest that physicians should consider the risk for prolonged *P*. *falciparum* infection in immigrant pregnant women and first-arrival immigrants even without recent travel to a country endemic for malaria. The prevalence of asymptomatic *P*. *falciparum* carriers in France or other northern countries is unknown but could be high with the increase in immigration. Public health authorities should be aware of the risk these persons represent for blood donations.
